# Anti-Osteoarthritic Effects of a Mixture of Dried Pomegranate Concentrate Powder, Eucommiae Cortex, and Achyranthis Radix 5:4:1 (*g*/*g*) in a Surgically Induced Osteoarthritic Rabbit Model

**DOI:** 10.3390/nu12030852

**Published:** 2020-03-22

**Authors:** Beom-Rak Choi, Su-Jin Kang, Jong-Lae Kim, Young-Joon Lee, Sae-Kwang Ku

**Affiliations:** 1 Department of Foodscience and Biotechnology, Sungkyunkwan University, Suwon-si, Gyeonggi-do 16419, Korea; brchoi@nutracore.co.kr; 2 Department of Histology and Anatomy, College of Korean Medicine, Daegu Haany University, Gyeongsan-si, Gyeongsangbuk-do 38610, Korea; vegonia1@hanmail.net; 3 The Medical Research Center for Herbal Convergence on Liver Disease, Daegu Haany University, Gyeongsan-si, Gyeongsangbuk-do 38610, Korea; 4 HLscience Co., Ltd. Uiwang-si, Gyeonggi-do 16004, Korea; kimjl@hlscience.com; 5 Department of Preventive Medicine, College of Korean Medicine, Daegu Haany University, Gyeongsan-si, Gyeongsangbuk-do 38610, Korea

**Keywords:** dried pomegranate juice concentrated powder, eucommiae cortex, achyranthis radix, PCP:EC:AR 5:4:1 (*g*/*g*) mixed formula, efficacy confirmation, surgically-induced osteoarthritic rabbits

## Abstract

In this study, we aimed to determine the synergistic effects of a formula consisting of dried pomegranate concentrate powder, Eucommiae Cortex, and Achyranthis Radix 5:4:1 (*g*/*g*) (PCP:EC:AR) in a surgically induced osteoarthritis (OA) rabbit model. PCP:EC:AR was orally administered once per day. Knee thickness, maximum extension of the knee joint, gross articular defect area, and the histopathological appearance of the cartilage were monitored, along with serum collagen type II C-telopeptide (CTX-II), cartilage oligomeric matrix protein (COMP), matrix metalloproteinase (MMP)-3, tumor necrosis factor (TNF)-α, interleukin (IL)-1β, and subchondral IL-1β and TNF-α levels. Roentgenographic images were also evaluated. PCP:EC:AR significantly inhibited the surgically induced increase in knee thickness, maximum extension of both knees, knee thickness after capsule exposure, gross femoral and tibial articular defect areas, loss of the knee joint area, serum and synovial COMP, CTX-II, and MMP expression, and synovial IL-1β, and TNF-α expression. In addition, surgically induced narrowing of the knee bones, loss of the joint area, cartilage damage, and osteophyte formation were reduced. PCP:EC:AR suppressed the surgically induced increases in the Mankin score, and subchondral IL-1β and TNF-α immunolabeled cell numbers. PCP:EC:AR exerted potent OA protective effects in a surgically induced OA rabbit model.

## 1. Introduction

Osteoarthritis (OA), a slowly progressive disease that causes functional disability, affects the majority of older people [1]. OA-related symptoms include joint pain, stiffness, limited movement with occasional effusion, inflammation, and structural changes [2,3]. The articular cartilage (AC) extracellular matrix (ECM) is composed of proteoglycan, collagen, and aggrecan [4,5]. Under normal conditions, collagen type 2 in the cartilaginous matrix contributes to the tensile strength of the tissue [6]. The composition and structure of the ECM changes with aging, and when the AC is exposed to mechanical damage [7]. Collagen has also been shown to change, from type 2 to type 1, in response to OA [8]. These changes in composition affect the mechanical stability of the ECM network. Inflammation is a critical factor in the development of OA [6,9]. The quality of the ECM is controlled by inflammatory cytokines and proteases. Various cytokines and chemokines, including tumor necrosis factor-α (TNF-α), interleukin-1β (IL-1β), IL-12, and IL-15, significantly increase the matrix-degrading proteins expression, including matrix metalloproteinases (MMPs) [10,11,12,13,14]. 

During tissue turnover, molecular biomarkers of OA are derived from tissue matrices released into fluids (i.e., blood, urine, or synovial fluid) [15,16]. They reflect bone or cartilage turnover and synovial metabolism, and can be used as an assessment of pathophysiological processes [17,18]. Higher baseline levels of collagen type II C-telopeptide (CTX-II) and cartilage oligomeric matrix protein (COMP) have been associated with the incidence of joint space narrowing, osteophytes, and knee OA [19,20]. MMP-3 levels have been also associated with the loss of volume [21]. Therefore, the value of CTX-II, COMP, and MMP-3 has been used as biomarkers of early-stage OA [15,16,22]. 

Pomegranate, which has been widely used in a therapeutic capacity [23], contains sugars, pectin, several tannins, and crude fiber [24]. Pomegranate seed juice and oil consist of anthocyanidins and flavonoids [25,26]. A large body of evidence shows that the pomegranate exerts antioxidant, anticarcinogenic, and anti-inflammatory effects [27,28,29,30]. In addition, molecular evidence supports that the pomegranate inhibits the IL-1β pathway in chondrocytes [31]. Eucommia ulmoides Oliver (EC) has been used in Korean medicine due to its antiviral, antihypertensive, and anti-inflammatory effects, as well as for its invigorating effects on the kidney and hepatoprotective action (without side-effects) [32,33,34]. In particular, EC protects cartilage by improving its metabolism, and inhibiting degradation of the ECM [35]. EC has been used as an anti-arthritic component in herbal formulations [36,37]. Achyranthes japonica Nakai (AR) has been commonly used to treat osteodynia of the knee and lumbar region, as well as spasm and flaccidity of the limbs, in traditional Korean medicine [38]. The bioactive compounds in AR, including inokosterone, ecdysterone, oleanolic acid, bisdesmoside, and saponins scavenge free radicals [39,40]. According to previous in vitro and in vivo studies, AR has anti-inflammatory, antioxidant, and anti-arthritic effects [41,42]. 

Mouse and rat models of OA have been developed [43]. These rodent models initially concerned the temporomandibular joint [44], but were then extended to encompass other synovial joints [45]. Furthermore, rodent models of chemically induced OA (using papain [46] or sodium iodoacetate [47], for example) have also been developed. The cartilage tissue of rats is insufficient for a multidisciplinary study; moreover, unlike humans, rats have open epiphyses. In contrast, due to their larger size, rabbit cartilage tissue is sufficient for cross-disciplinary studies, and the surgically induced OA shows a similar gross appearance between the rabbit and human knee. Thus. rabbit models, including anterior cruciate ligament transection and partial meniscectomy models [48,49], have been used to assess the anti-osteoarthritic potential of various drugs [50,51]. To successfully examine the potential effects of drugs against OA, selection of an appropriate animal model is important. In this experiment, we determined the synergistic effects of a mixed formula, consisting of dried pomegranate concentrate powder (PCP), Eucommiae Cortex (EC), and Achyranthis Radix (AR) 5:4:1 (*g*/*g*) (PCP:EC:AR) in a surgically induced OA rabbit model. We aimed to determine whether there are species-specific anti-OA effects, where we previously demonstrated anti-OA effects in a surgically induced OA rat model. 

Synergistic increases in the biological activities of individual components can often be achieved using mixtures [52,53]. Therefore, we expected that a mixture of PCP, EC, and AR would show high efficacy against OA. The PCP:EC:AR mixture has shown anti-inflammatory activity in rat chondrocytes [54]. We previously demonstrated that PCP:EC:AR exerts greater anti-OA effects in a surgically induced OA rat model than each component individually. In this study, our objective was to confirm the anti-OA potential of PCP:EC:AR in a surgically induced OA rabbit model [48,49] to confirm its therapeutic potential against OA. 

PCP:EC:AR mixtures were orally administered to rabbits after transecting the anterior cruciate ligament and performing partial medial meniscectomy. Knee thickness, maximum extension of the knee joint, gross articular defect area, serum CTX-II, COMP, and MMP-3 levels, synovial fluid CTX-II, COMP, IL-1β, MMP-3, and tumor necrosis factor (TNF)-α levels, the histopathological appearance of cartilage, Mankin score, thickness of the cartilage, number of chondrocytes in tibial and femoral articular regions, and subchondral IL-1β and TNF-α immunoreactive cell numbers were monitored as histomorphometric indices. Roentgenographic images were also assessed.

## 2. Materials and Methods 

### 2.1. Animals and Husbandry

Sixty healthy male New Zealand White rabbits (11 weeks old, body weights ranged from 1.8–2.2 kg upon receipt; Shinyang Rabbit Farm, Hwasung, Korea) were used after 1 week of acclimatization to the laboratory environment. Four animals per stainless wire cages were allocated in a 20–25 °C temperature- and 45%–55% humidity-controlled room. There were 50 OA-induced rabbits; the remaining 10 rabbits were sham-operated controls. Five weeks after the OA operation, eight rabbits in each group were assessed in terms of knee thickness and body weight. Animals were maintained based on the national regulations of the usage and welfare of laboratory animals, and approved by the Institutional Animal Care and Use Committee in Kyungpook National University (Daegu, Korea) prior to the experiment [Approval No. KNU2015-0144].

### 2.2. Preparation and Administration of Test Substances

PCP (ASYA Meyve Suyu ve Gıda San. A.Ş., Ankara, Turkey) contained 1.15 mg/g ellagic acid, EC contained 1.58 mg/g pinoresinol diglucoside, and AR contained 0.26 mg/g ecdysterone as active components, respectively (Health Love Ltd., Anyang, Korea). PCP:EC:AR doses of 50, 100, and 200 mg/kg represented the high-, medium-, and low-dose conditions, respectively, as in our previous study on dose-dependent efficacy in a surgically induced OA rat model [55]. The PCP:EC:AR mixed formula (50, 100, and 200 mg/kg) was administered orally (once a day for 56 days, beginning 5 weeks after OA surgery) in a volume of 5 mL/kg distilled water using a gastric tube attached to a 20 mL syringe. Diclofenac (1 mL/kg; 2 mg/mL sterilized saline) was injected subcutaneously into the dorsal skin with a 26-G needle, in accordance with our previous rat studies [52,56]. The PCP:EC:AR mixed formula was prepared by directly dissolving the PCP, EC, and AR using distilled water. Equal volumes of vehicle were orally administered to sham and OA control rabbits.

### 2.3. OA Induction by Anterior Cruciate Ligament Transection and Partial Medial Meniscectomy

Surgery was performed in rabbits to induce OA by anterior cruciate ligament transection and partial medial meniscectomy [48,49]. All animals were anesthetized by an intraperitoneal injection of 25 mg/kg Zoletil (Zoletile 50; Virbac Lab., Carros, France), and were maintained on 1%–1.5% isoflurane in a mixture of 70% N_2_O and 28.5% O_2_ using an inhalation anesthesia apparatus (Surgivet, Waukesha, WI, USA) and a rodent ventilator (Model 687, Harvard Apparatus, Cambridge, UK). The OA treatment group underwent open surgery of the left knee joint, which involved anterior cruciate ligament transection and partial medial meniscectomy via an incision on the medial aspect of the joint capsule, anterior to the medial collateral ligament. The incision was closed in two layers. The joint capsule was sutured independently from the peripheral tissues using dissolvable 5-0 Vicryl sutures, and the skin was closed with interrupted silk sutures. This treatment was used to induce OA. Conversely, the right knee joint was used as the contralateral treatment. The second group of rabbits underwent a sham operation, in which a similar incision was made in the joint capsule, but the anterior cruciate ligament was not transected and no partial medial meniscectomy was performed (according to established methods) [48,49].

### 2.4. Body Weight Measurements

Body weight was measured at the time of OA surgery, at the time of initial treatment administration, and 7, 14, 21, 28, 35, 42, 49, 55, and 56 days thereafter using an electronic balance (Precisa Instrument, Dietikon, Switzland). All animals were fasted overnight (water was not) before weighing to reduce the effects of feeding on the measurements. 

### 2.5. Knee Thickness Measurements

The thicknesses of the OA-operated hind knees were measured using digital calipers (Mytutoyo, Tokyo, Japan), and after exposing the joint capsule at euthanization (to reduce the influence of surrounding tissues, according to our previously established method) [52,56]. Changes in knee thickness after OA surgery were calculated. 

### 2.6. Measurement of Maximum Knee Extension 

The OA-operated knees were dissected from the coxofemoral region to the ankle region, leaving the articular capsule intact. After dissection, the maximum extension of each knee was measured according to previous methods [52,56,57], with 0° corresponding to the maximum possible extension. All measurements were performed by the same veterinarian to minimize bias.

### 2.7. Measurement of Gross Articular Defect Area

After measuring the maximum extensor angle, the femoral and tibial articular surfaces were exposed, and the OA-induced defect area (% of defect cartilages) was measured using a computer-assisted image analysis program (*i*Solution FL ver 9.1; IMT *i*-solution Inc., Vancouver, Quebec, Canada) after staining with black India ink (Office center, Seoul, Korea).

### 2.8. Measurement of Serum COMP, CTX-II, and MMP-3 Levels

Approximately 10 mL of whole blood was collected from the vena cava at euthanization, and the serum was separated by centrifugation at 3000 rpm for 10 min. Serum concentrations of COMP, CTX-II, and MMP-3 were measured using sandwich enzyme-linked immunosorbent assays (ELISAs) in accordance with previous reports [15,16]. A rabbit COMP ELISA kit (MBS721182, MyBioSource, San Diego, CA, USA), rabbit CTX-II ELISA kit (MBS705896, MyBioSource, San Diego, CA, USA) and rabbit MMP-3 ELISA kit (MBS2502146, MyBioSource, San Diego, CA, USA) were used in this measurement. All ELISA procedures were performed according to the manufacturer’s instructions, except for the dilution ratio. Standards and samples (100 μL) were added to the appropriate wells and incubated for 2 h at 37 °C. The wells were aspirated, and a 1× biotin-labeled antibody (100 μL) was added to each well, followed by incubation for 1 h at 37 °C. The wells were then aspirated again, washed three times, and incubated with 1× horseradish peroxidase-conjugated avidin (100 μL) for 1 h at 37 °C. 3,3′,5,5′-Tetramethylbenzidine substrate (90 μL) was added to each well, followed by incubation for 15–30 min at 37 °C. Stop solution (50 μL) was then added to each well, and absorbance was measured at 450 nm using a microplate reader (Tecan, Männedorf, Switzerland). 

### 2.9. Measurement of Synovial COMP, CTX-II, MMP-3, IL-1β, and TNF-α Levels

Synovial fluid was collected from the operated knee before euthanization and centrifuged at 12,500 rpm for 10 min at 4 °C. Synovial concentrations of COMP, CTX-II, MMP-3, IL-1β, and TNF-α were measured using ELISAs in accordance with previous reports [15,16,49,58], using methods similar to those described for the serum measurements. A rabbit COMP ELISA kit (MBS721182, MyBioSource, San Diego, CA, USA), rabbit CTX-II ELISA kit (MBS705896, MyBioSource, San Diego, CA, USA), rabbit MMP-3 ELISA kit (MBS2502146, MyBioSource, San Diego, CA, USA), rabbit IL-1β ELISA kit (LS-F23299, LSBio Seattle, WA, USA) and rabbit TNF-α ELISA kit (MBS2500169, MyBioSource, San Diego, CA, USA) were used.

### 2.10. Histology

Knee joint parts were obtained while preserving the joint capsules, fixed in 10% neutral buffered formalin, and then decalcified in decalcifying solution [24.4% formic acid, and 0.5N sodium hydroxide] for 5 days (mixed decalcifying solution was changed once a day for 5 days). Each knee joint was longitudinally trimmed, embedded in paraffin, and sectioned (3–4 μm) using a tungsten bladder equipped with n Polycut microtome (Model RM2255, Leica, Wetzlar, Germany). Cartilaginous tissue was stained with Safranin O [52,56,59]. The histological results were analyzed using a light microscope (Model Eclipse 80*i*, Nikkon, Tokyo, Japan) by a researcher who was blinded to the group assignments.

### 2.11. Immunohistochemistry

Immunoreactivity of the proinflammatory cytokines, IL-1β and TNF-α [60,61], was assessed. Briefly, endogenous peroxidase activity was blocked by incubating tissue sections in methanol and 0.3% H_2_O_2_ for 30 min, and non-specific binding of immunoglobulin was blocked with horse serum blocking solution for 1 h in a humidity chamber, after epitope retrieval by pretreatment of trypsin (Sigma-Aldrich, St. Louise, MO, USA) and 2 N HCl on unstained sections, as described previously [52,56]. The sections were incubated with primary antisera overnight at 4 °C in a humidity chamber, and then with biotinylated universal secondary antibody and ABC reagents for 1 h at room temperature in a humidity chamber. Finally, the sections were reacted with a peroxidase substrate kit for 3 min at room temperature. 

### 2.12. Histomorphometry

Femoral and tibial AC in the knees was evaluated using the Mankin scoring system and Safranin O staining [52,56]. In the Mankin scoring system, higher scores indicate more severe OA (Semiquantative scores; Max = 12). The entire histological evaluation was performed by the same pathologist. In addition, the thickness of the tibial and femoral AC (μm/cartilage), and the numbers of femoral and tibial AC chondrocytes (cells/mm^2^), were measured in longitudinally trimmed samples using a computer-based automated image analyzer according to our previously established method [52,56,62,63]. The histopathologist was blinded to the group assignments. Cells with more than 20% immunoreactivity, as indicated by IL-1β and TNF-α antibodies, were regarded as positive [52,56], and the numbers of immunoreactive cells were counted separately in the femoral and tibial subchondral regions using an automated digital image analyzer (cells/mm^2^). One histological region of femoral and tibial AC was assessed in each rabbit (a total of eight fields in each group).

### 2.13. Statistical Analyses

All values are expressed as mean ± SD of eight rabbits. Multiple comparison tests for different dose groups were conducted. Homogeneity of variance was performed using the Levene test. If the Levene test indicated an equal variance, the obtained data were analyzed using a one way ANOVA test with a least-significant differences (LSD) multi-comparison test, as a post-hoc test. If the homogeneity of variance was violated, the Kruskal-Wallis H test was conducted. When a rejection of the null hypothesis is observed, the Mann-Whitney U (MW) test was performed. Statistical analyses were performed using SPSS (Release 14.0K, SPSS Inc., Chicago, IL, USA). In addition, the changes in percentage between sham and OA control rabbits were calculated to observe the severities of induced OA signs, and the changes of percentage as compared with OA control and test substance administered rabbits were also calculated to help the understanding of the efficacy of test materials. 

## 3. Results

### 3.1. Effects of Treatment on Body Weight and Knee Thickness

At 35 days after surgery, the average weight of eight rabbits in each of the sham and OA groups was 2.39 ± 0.15 and 2.38 ± 0.12 kg, respectively. No significant difference in body weight was observed between the two groups (Table 1). 

**Figure 1:**
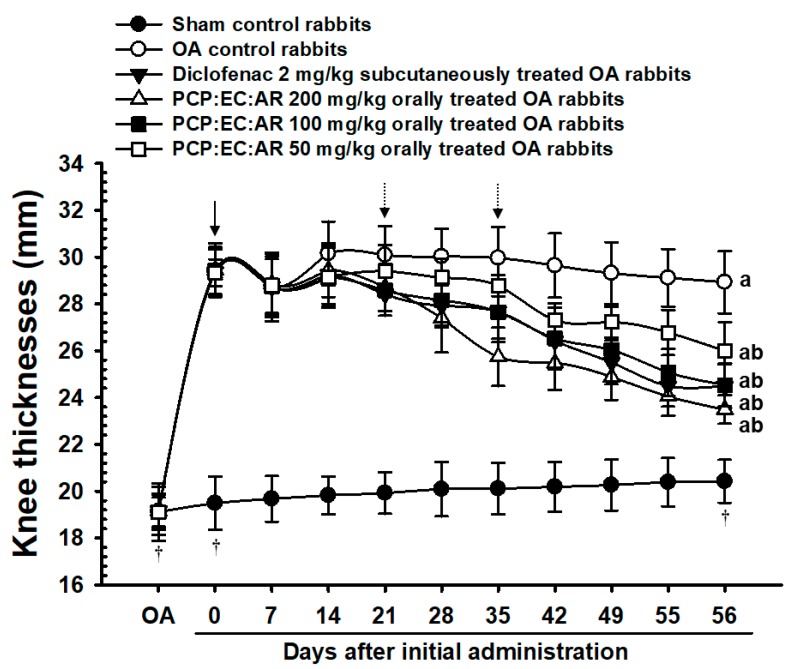
Knee thickness changes in sham-operated or OA rabbits. Significant (*p* < 0.01) increases of knee thicknesses were demonstrated in OA control rabbits from 35 days after OA (one day before initial administration) as compared with sham control rabbits, throughout the whole experiment periods in the present study (arrow). Significant (*p* < 0.01 or *p* < 0.05) decreases of knee thicknesses were demonstrated in diclofenac, PCP: EC:AR mixed formula 100 and 200 mg/kg treated rabbits from 21 days after initial administration as compared with OA control rabbits, and from 35 days after initial administration in PCP:EC:AR mixed-formula 50 mg/kg treated rabbits as compared with OA control rabbits, respectively (dot arrows). Values are expressed as mean ± S.D. of eight rabbits, mm. OA = Osteoarthritis; AR = Aqueous extracts of Achyranthis Radix; EC = Aqueous extracts of Eucommiae Cortex; PCP = Dried Pomegranate Juice Concentrated Powder. OA means at OA surgery; 0 means at start of administration, at 35 days after OA operation; 56 means 56 days after initiation of administration, at euthanization. All animals were fasted overnight before OA surgery, the first administration, and euthanization, respectively (†). ^a^ *p* < 0.01 as compared with sham control; ^b^ *p* < 0.01 as compared with OA control.

Eight rabbits from each group exhibiting increased knee thickness at 35 days after the OA operation were compared with sham control rabbits (arrow). The OA control rabbits showed increased knee thickness compared with the sham control rabbits, while the PCP:EC:AR (100 and 200 mg/kg)-treated rabbits showed an decrease in knee thickness beginning 21 days after initial administration of the treatment compared with the OA control rabbits, and from 35 days after initial administration in the PCP:EC:AR mixed-formula 50 mg/kg treated rabbits as compared with OA control rabbits, respectively (dot arrows). PCP:EC:AR treatment (50, 100, and 200 mg/kg) led to a dose-dependent decrease in knee thickness (Table 2, Figure 1).

### 3.2. Effects of Treatment on Knee Thickness after Capsule Exposure

**Figure 2:**
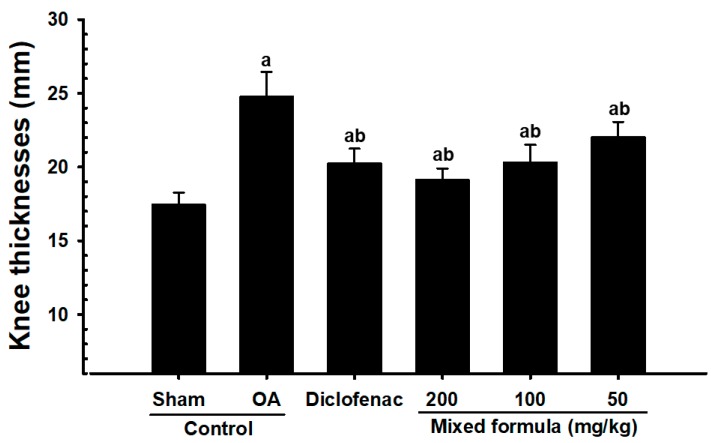
Knee thickness after capsule exposure in sham-operated or OA rabbits. Values are expressed as mean ± S.D. of eight rabbits, mm. OA = Osteoarthritis; AR = Aqueous extracts of Achyranthis Radix; EC = Aqueous extracts of Eucommiae Cortex; PCP = Dried Pomegranate Juice Concentrated Powder. ^a^ *p* < 0.01 as compared with sham control; ^b^ *p* < 0.01 as compared with OA control.

Capsule-exposed knee thicknesses were greater in OA control rabbits than in sham control rabbits. However, significantly decreased knee thickness was observed after exposing the capsule in all PCP:EC:AR (50, 100, and 200 mg/kg)-treated rabbits compared with the OA control rabbits, in a dose-dependent manner (Figure 2).

### 3.3. Effects of Treatment on Maximum Knee Extension

**Figure 3:**
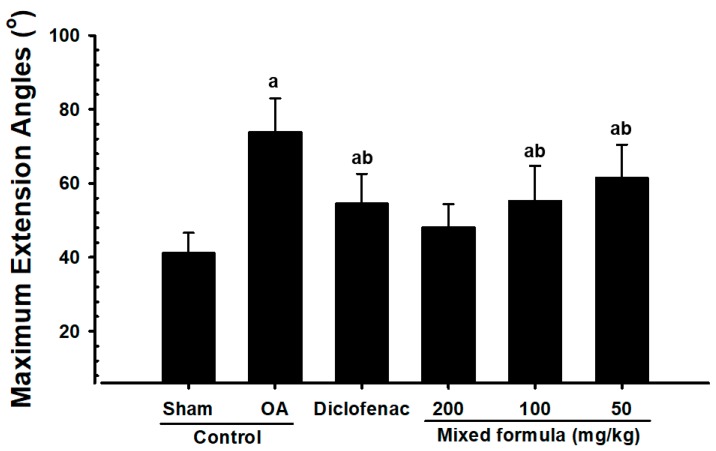
Maximum knee extension angles in sham-operated or OA rabbits. Values are expressed as mean ± S.D. of eight rabbits, in degrees (°). AR = Aqueous extracts of Achyranthis Radix; OA = Osteoarthritis; EC = Aqueous extracts of Eucommiae Cortex; PCP = Dried Pomegranate Juice Concentrated Powder. ^a^ *p* < 0.01 as compared with sham control; ^b^ *p* < 0.01 as compared with OA control.

Significant increases in maximum knee extension were observed in OA control rabbits compared with the sham control rabbits. However, the maximum extension of the knee decreased significantly in the PCP:EC:AR (50, 100, and 200 mg/kg)-treated rabbits compared with the OA control rabbits, in a dose-dependent manner (Figure 3).

### 3.4. Changes in Gross Articular Defect Areas

**Figure 4:**
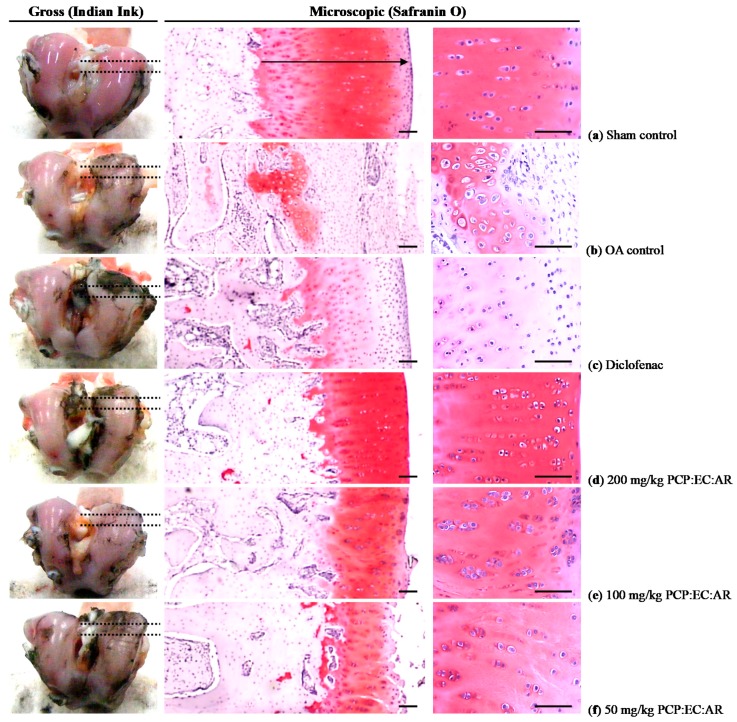
Representative gross and general histological images of the femur articular surface cartilages, taken from sham-operated or OA rabbits. (**a**) Sham vehicle control (sham-operated and distilled water orally administered rabbits); (**b**) OA control (OA-surgery and distilled water orally administered rabbits); (**c**) Diclofenac (OA-surgery and diclofenac sodium 2 mg/kg subcutaneously treated rabbits); (**d**) PCP:EC:AR mixed-formula 200 mg/kg orally administrated OA rabbits; (**e**) PCP:EC:AR mixed formula 100 mg/kg orally administrated OA rabbits; (**f**) PCP:EC:AR mixed formula 50 mg/kg orally administrated OA rabbits. OA = Osteoarthritis; AR = Aqueous extracts of Achyranthis Radix; EC = Aqueous extracts of Eucommiae Cortex; PCP = Dried Pomegranate Juice Concentrated Powder. Arrows indicated the thicknesses of femur articular cartilage; Dot lines indicated the regions of histological observations. Scale bars = 200 µm.

**Figure 5:**
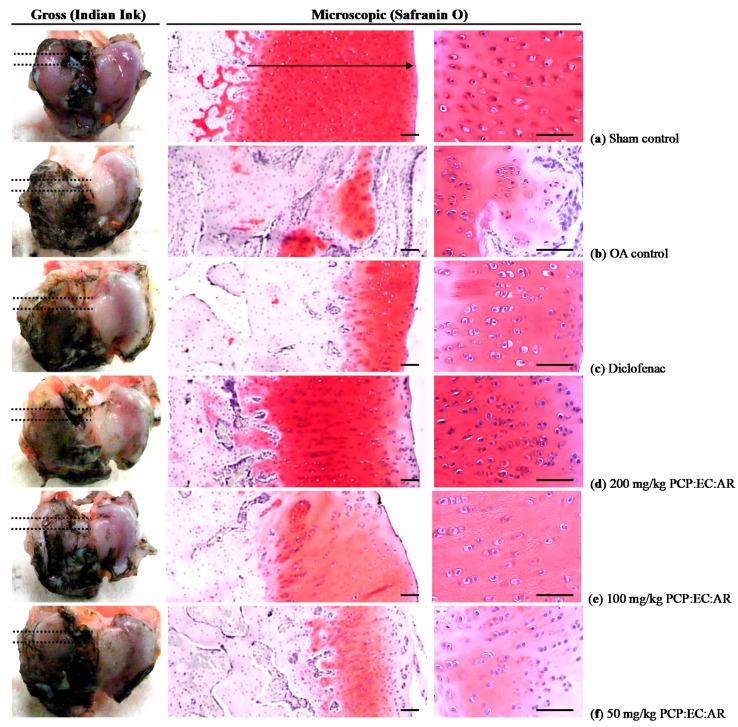
Representative gross and general histological images of the tibia articular surface cartilages, taken from sham-operated or OA rabbits. (**a**) Sham vehicle control (sham-operated and distilled water orally administered rabbits); (**b**) OA control (OA-surgery and distilled water orally administered rabbits); (**c**) Diclofenac (OA-surgery and diclofenac sodium 2 mg/kg subcutaneously treated rabbits); (**d**) PCP:EC:AR mixed formula 200 mg/kg orally administrated OA rabbits; (**e**) PCP:EC:AR mixed formula 100 mg/kg orally administrated OA rabbits; (**f**) PCP:EC:AR mixed formula 50 mg/kg orally administrated OA rabbits. OA = Osteoarthritis; AR = Aqueous extracts of Achyranthis Radix; EC = Aqueous extracts of Eucommiae Cortex; PCP = Dried Pomegranate Juice Concentrated Powder. Arrows indicated the thicknesses of tibia articular cartilage; Dot lines indicated the regions of histological observations. Scale bars = 200 µm.

**Figure 6:**
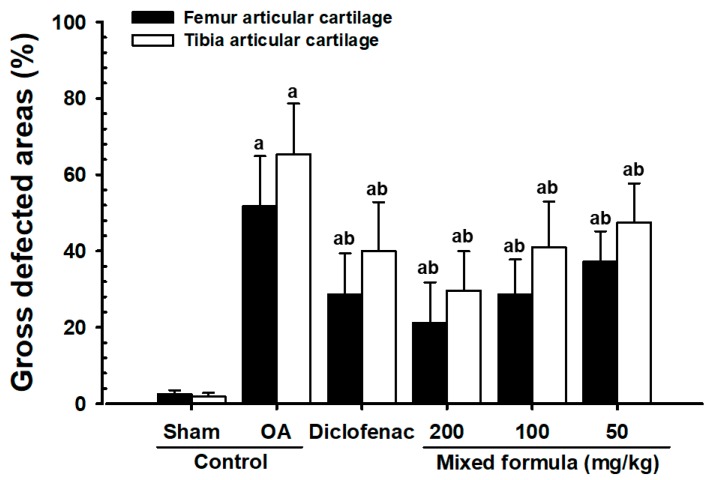
Changes on the femur and tibia articular cartilage defect areas in sham-operated or OA rabbits. Values are expressed mean ± S.D. of eight rabbits, %. OA = Osteoarthritis; AR = Aqueous extracts of Achyranthis Radix; EC = Aqueous extracts of Eucommiae Cortex; PCP = Dried Pomegranate Juice Concentrated Powder. ^a^ *p* < 0.01 as compared with sham control; ^b^ *p* < 0.01 and ^c^ *p* < 0.05 as compared with OA control.

The femoral and tibial gross articular defect areas increased in the OA control, compared with sham control rabbits. In contrast, all PCP:EC:AR (50, 100, and 200 mg/kg)-treated rabbits showed dose-dependent decreases in gross femoral and tibial AC defect areas (Figure 4, Figure 5 and Figure 6).

### 3.5. Changes in Serum COMP, CTX-II, and MMP-3 Levels

Serum COMP, CTX-II, and MMP-3 levels were higher in the OA control rabbits than in the sham control rabbits. However, PCP:EC:AR (50, 100, and 200 mg/kg) decreased serum COMP, CTX-II, and MMP-3 levels compared with those in OA control rabbits, in a dose-dependent manner (Table 3). 

### 3.6. Changes in Synovial COMP, CTX-II, MMP-3, IL-1β, and TNF-α Levels

Synovial COMP, CTX-II, MMP-3, IL-1β, and TNF-α levels were higher in OA control rabbits than in sham control rabbits. However, PCP:EC:AR (50, 100, and 200 mg/kg) decreased synovial COMP, CTX-II, MMP-3, IL-1β, and TNF-α levels compared with those of OA control rabbits, in a dose-dependent manner (Table 4). 

### 3.7. Changes in Femoral and Tibial AC Mankin Scores

Marked increases in surface cartilage damage, a decreased number of chondrocytes, clone formation, and decreased Safranin O staining intensity were detected in the femoral and tibial AC of OA control rabbits. Consequently, the femoral and tibial AC Mankin scores were higher in OA control rabbits than in sham control rabbits. However, the femoral and tibial AC Mankin scores were lower in all PCP:EC:AR treatment groups (50, 100, and 200 mg/kg) than in OA control rabbits, in a dose-dependent manner (Table 5, Figure 4 and Figure 5).

### 3.8. Histopathological Changes in the Femoral and Tibial AC

Femoral AC thickness decreased significantly in OA control rabbits compared with sham control rabbits. In contrast, significant dose-dependent increases in femoral AC thickness were observed in all PCP:EC:AR treatment groups (50, 100, and 200 mg/kg) compared with OA control rabbits.

Tibial AC thickness decreased significantly in OA control rabbits. In contrast, tibial AC thickness increased significantly in all PCP:EC:AR (50, 100, and 200 mg/kg)-treated rabbits compared with OA control rabbits, in a dose-dependent manner (Table 6, Figure 4).

A significant decrease in the number of femoral AC chondrocytes was detected in OA control rabbits compared with sham control rabbits; however, a significant dose-dependent increase was observed in all PCP:EC:AR treatment groups (50, 100, and 200 mg/kg) compared with OA control rabbits (Table 6, Figure 4).

Fewer tibial AC chondrocytes were observed in the OA control rabbits than in the sham control rabbits; however, the number of tibial AC chondrocytes increased significantly in all PCP:EC:AR treatment groups (50, 100, and 200 mg/kg) compared with the OA control rabbits, in a dose-dependent manner (Table 6, Figure 5).

### 3.9. Effects of Treatment on IL-1β-Immunoreactive Cells in Femoral and Tibial Subchondral Regions

**Figure 7:**
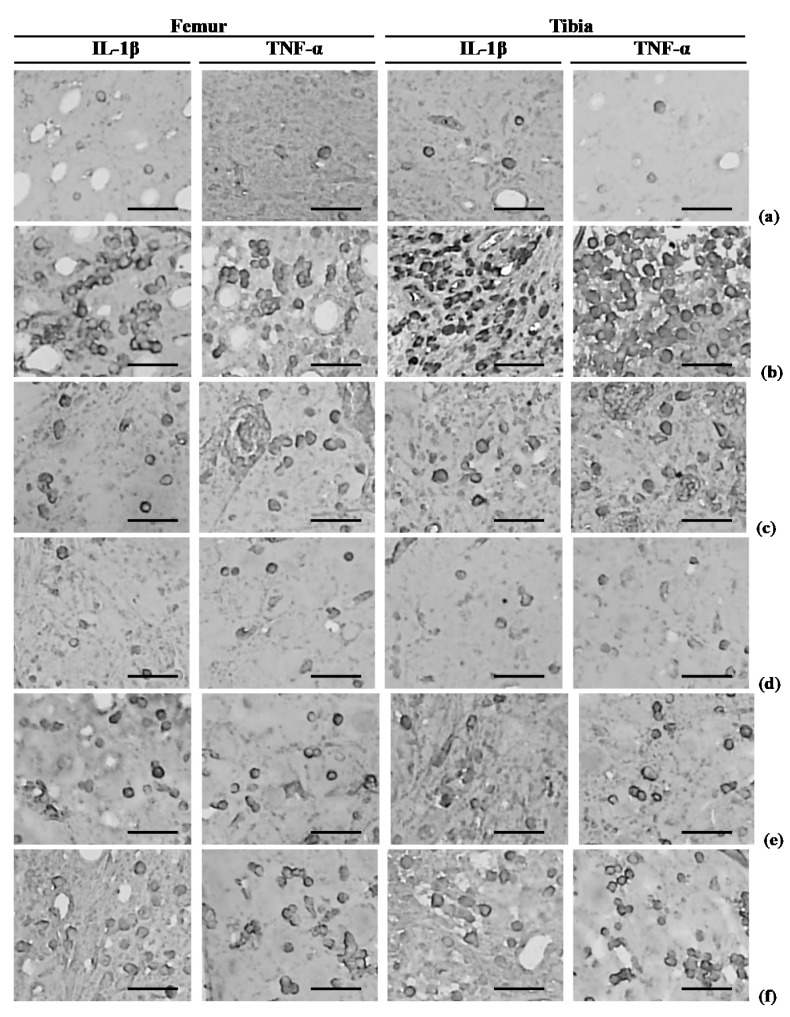
Representative immunohistochemistrical images of the femur and tibia subchondral IL-1β and TNF-α-immunoreactive cells, taken from sham-operated or OA rabbits. (**a**) Sham vehicle control (sham-operated and distilled water orally administered rabbits); (**b**) OA control (OA-surgery and distilled water orally administered rabbits); (**c**) Diclofenac (OA-surgery and diclofenac sodium 2 mg/kg subcutaneously treated rabbits); (**d**) PCP:EC:AR mixed formula 200 mg/kg orally administrated OA rabbits; (**e**) PCP:EC:AR mixed formula 100 mg/kg orally administrated OA rabbits; (**f**) PCP:EC:AR mixed formula 50 mg/kg orally administrated OA rabbits. OA = Osteoarthritis; AR = Aqueous extracts of Achyranthis Radix; EC = Aqueous extracts of Eucommiae Cortex; PCP = Dried Pomegranate Juice Concentrated Powder; ABC = Avidin-biotin complex; IL = Interleukin; TNF = Tumor necrosis factor. All ABC immunostain. Scale bars = 100 µm.

Significant increases in the numbers of femoral and tibial subchondral IL-1β-immunopositive cells were detected in OA control rabbits. However, significant dose-dependent decreases in femoral and tibial subchondral IL-1β-immunoreactive cells were observed in all PCP:EC:AR (50, 100, and 200 mg/kg)-treated rabbits compared with OA control rabbits (Table 6, Figure 7).

### 3.10. Effects of Treatment on TNF-α-Immunoreactive Cells in Femoral and Tibial Subchondral Regions

The numbers of femoral and tibial subchondral TNF-α-immunopositive cells increased in OA control rabbits, whereas they decreased in all PCP:EC:AR treatment groups (50, 100, and 200 mg/kg) in a dose-dependent manner (Table 6, Figure 7).

## 4. Discussion

The anti-OA potential of PCP:EC:AR was demonstrated in a surgically induced OA rabbit model [48,49]. The anti-OA effects of PCP:EC:AR were similar to those seen in rat and mouse models of OA; thus, PCP:EC:AR effectively relieves OA symptoms in various animal models. The PCP:EC:AR formula used herein was informed by our previous in vitro [54,64] and in vivo [55,65] experiments on OA rats. 

OA is a debilitating disease that affects the AC and subchondral bone. Inflammation is closely involved in all steps of OA progression, where cartilage damage triggers prostaglandin and leukotriene activation by cyclooxygenase-2 and 5-lipoxygenase, respectively. In addition, the inflammatory factors TNF-α and IL-1β contribute to OA progression. The inflammatory response results in swelling of the tissues surrounding the joints, in association with increased joint thickness, irregular bone growth, and osteophyte formation [66,67,68]. In the present study, OA-induced rabbits exhibited significantly increased knee thickness, decreases in AC thickness of the femur and tibia and the number of chondrocytes, significantly increased synovial TNF-α and IL-1β levels, and significantly higher numbers of femoral and tibial subchondral IL-1β and TNF-α-immunolabeled cells. However, these OA-related signs of inflammation in OA rabbits were dose-dependently and significantly inhibited by 56 days of administration with PCP:EC:AR (50, 100, and 200 mg/kg). These findings represent reliable evidence that a PCP:EC:AR mixed formula has favorable anti-inflammatory effects on surgically induced OA rabbits.

X-ray images have been widely used to detect loss of cartilage erosion, joint region, and osteophyte formation during the development of OA [62,69]. Thus, anti-OA agents should inhibit cartilage damage on X-ray images to indicate efficacy [49,70]. In our experiment, articular space narrowing, loss of the femoral and tibial AC erosion, joint region, and osteophyte formation were detected on roentgenographic images of the OA control rabbits. In addition, significant increments of gross articular defect areas in the femur and tibia were also detected in OA control rabbits. However, these changes were inhibited dose-dependently and significantly by 56 days of continuous treatment with PCP:EC:AR (50, 100, and 200 mg/kg), similar to the effects of subcutaneous diclofenac (2 mg/kg) treatment. These results show that PCP:EC:AR exerted anti-OA effects in a surgically induced OA rabbit model (Supplementary Data, Figure S1).

Fibrosis and inflammation have been linked to OA progression, and anti-fibrotic drugs have shown promise in the clinical treatment of OA. The anti-fibrotic and anti-inflammatory drug pirfenidone shows efficacy against kidney and lung fibrosis. Pirfenidone may encourage tissue repair and prevent progression of post-traumatic OA [71]. The fibrosis that occurs in OA due to chronic inflammation limits joint movement, where stiffness of the joints is a major symptom of OA. Joint stiffness is evaluated based on the maximum extension of the joint, where a 0 degree corresponds to the maximum extension, that is, better knee function [52,56,57,62]. Thus, the significant increases in maximum extension of the knees of our OA control rabbits indicate that OA was well-induced. However, these OA-related increases in maximum knee extension were also reduced dose-dependently and significantly by 56 days of continuous treatment with all three doses (50, 100, and 200 mg/kg) of PCP:EC:AR. These findings represent direct evidence that PCP:EC:AR inhibits joint stiffness induced by OA.

COMP, a thrombospondin gene family member, is widely expressed in the ECM of AC [72,73]. COMP binds collagen types Ⅰ, II, and Ⅳ, and is involved in the regulation of chondrocytes [74]. Kuhne et al. [75] reported that the serum COMP level in serum increases in patients after traumatic knee injury. COMP may be instructive regarding cartilage degradation status, and has been used as a marker of OA [15,16,76]. This protein is a constituent of AC, but is also synthesized by tendon synoviocytes and fibroblasts [76,77]. Marked increases in synovial and serum COMP levels were detected in our OA control rabbits. However, significant dose-dependent decrements in synovial and serum COMP levels were detected in the PCP:EC:AR (50, 100, and 200 mg/kg)-treated rabbits, indicating a favorable AC-preserving effect.

Type II collagen is found mainly in cartilage, of which it is a major structural component [78]. The normal cartilage matrix consists of more collagen type II than collagen type I [6,79,80]. Collagen type II shows strong interactions with proteoglycans, so the ECM network is affected by collagen type II content. Collagen type II content decreases during the development of OA, such that ECM integrity is inevitably reduced. Hence, measurements of constituents of this protein, such as CTX-II, may have utility as an index of cartilage degradation [76]. Increased CTX-II has also proven valuable as a biomarker for early OA [15,16]. In the present study, noticeable increases in serum and synovial CTX-II levels were observed in OA control rabbits, which corresponded well to the increases in COMP. However, dose-dependent significant decreases in synovial and serum CTX-II levels were detected in the PCP:EC:AR 5:4:1 (50, 100, and 200 mg/kg)-treated rabbits. These findings represent additional indirect evidence that the PCP:EC:AR mixture has favorable AC-preserving effects. 

MMPs are involved in degradation of the ECM (mainly proteoglycans) [81], although their expression can be suppressed by metalloproteinase inhibitors [82]. In particular, the MMP-3 level is a reliable predictor of cartilage loss during the development of OA [21]. MMP-3 increases in the synovium and chondrocytes in surgically induced OA rabbit models [83]. Davidson et al. reported that less severe OA in joints was observed in MMP-3 knockout mice [84,85]. In the present study, significant increases in serum and synovial MMP-3 levels were observed in OA control rabbits, but these increases were inhibited dose-dependently and significantly by 56 days of continuous treatment of 50, 100, and 200 mg/kg PCP:EC:AR. These findings represent additional evidence that the anti-OA activity of PCP:EC:AR may be mediated by MMP-3 inhibition.

Mankin’s 14-point histopathological grading system of cartilage deterioration is based on cartilage surface damage, the number of chondrocytes, and Safranin O staining. In this study, normal cartilage was scored as 0; the most severe case of cartilage deterioration had a score of 12 [52,56,57,86]. In addition, decreases in AC thickness and the number of chondrocytes have been consistently detected in animal models of OA [52,56]. In the present experiment, these increments induce by OA in Mankin scores of femoral and tibial AC, and the decreases in chondrocyte numbers and cartilage thickness, were inhibited dose-dependently and significantly by 56 days of continuous treatment with PCP:EC:AR (50, 100, and 200 mg/kg) compared with those of OA control rabbits. These findings indicate that the PCP:EC:AR mixture has chondrocyte-protective effects in surgically induced OA rabbits, at least under the conditions of the present experiment.

## 5. Conclusions

PCP:EC:AR (50, 100, and 200 mg/kg) consistently showed significant dose-dependent activity against OA in a rabbit model, similar to subcutaneous diclofenac (2 mg/kg) treatment. Taken together, the results indicated that the PCP:EC:AR mixed formula is a promising novel agent for relieving OA symptoms.

## Figures and Tables

**Table 1:** Body weight gains in sham-operated or OA rabbits.

Periods Groups	Body Weights (kg) at				Body Weight Gains (g)	
	OA [A] *	First Treatment [B] *	Last Treatment	Euthanize [C] *	OA Induced [B-A]	Treatment [C-B]
Controls						
Sham	1.98 ± 0.09	2.39 ± 0.15	2.66 ± 0.12	2.50 ± 0.13	0.41 ± 0.08	0.11 ± 0.10
OA	1.96 ± 0.11	2.39 ± 0.14	2.66 ± 0.22	2.51 ± 0.20	0.43 ± 0.09	0.13 ± 0.20
Diclofenac	1.96 ± 0.11	2.38 ± 0.13	2.68 ± 0.13	2.54 ± 0.14	0.41 ± 0.06	0.16 ± 0.18
Mixed formula—PCP:EC:AR						
200 mg/kg	1.99 ± 0.08	2.38 ± 0.07	2.65 ± 0.18	2.55 ± 0.18	0.39 ± 0.10	0.18 ± 0.21
100 mg/kg	1.96 ± 0.11	2.38 ± 0.13	2.69 ± 0.15	2.54 ± 0.18	0.41 ± 0.04	0.16 ± 0.12
50 mg/kg	1.96 ± 0.13	2.36 ± 0.17	2.73 ± 0.12	2.58 ± 0.15	0.40 ± 0.05	0.21 ± 0.21

Values are expressed mean ± SD of eight rabbits, kg. OA = Osteoarthritis; AR = Aqueous extracts of Achyranthis Radix; EC = Aqueous extracts of Eucommiae Cortex; PCP = Dried Pomegranate Juice Concentrated Powder. * All animals were overnight fasted.

**Table 2:** Knee thickness changes in sham-operated or OA rabbits.

Periods Groups	Knee Thicknesses (mm) at		Changes after OA Surgery (mm) [B-A]
	OA [A] *	Euthanize [B] *	
Controls			
Sham	19.11 ± 1.23	20.43 ± 0.92	1.31 ± 0.67
OA	19.14 ± 0.76	28.93 ± 1.33 ^a^	9.79 ± 1.33 ^a^
Diclofenac	19.15 ± 0.71	24.49 ± 0.92 ^ab^	5.34 ± 1.20 ^ab^
Mixed formula—PCP:EC:AR			
200 mg/kg	19.16 ± 1.02	23.49 ± 0.61 ^ab^	4.33 ± 1.01 ^ab^
100 mg/kg	19.10 ± 0.76	24.54 ± 0.92 ^ab^	5.44 ± 1.24 ^ab^
50 mg/kg	19.11 ± 0.63	26.00 ± 1.22 ^ab^	6.89 ± 1.02 ^ab^

Values are expressed mean ± SD of eight rabbits, mm. OA = Osteoarthritis; AR = Aqueous extracts of Achyranthis Radix; EC = Aqueous extracts of Eucommiae Cortex; PCP = Dried Pomegranate Juice Concentrated Powder. * All animals were overnight fasted. ^a^ *p* < 0.01 as compared with sham control; ^b^ *p* < 0.01 as compared with OA control.

**Table 3:** Serum COMP, CTX-II and MMP-3 levels in sham-operated or OA rabbits.

Items Groups	Serum Levels (μg/L)		
	COMP	CTX-II	MMP-3
Controls			
Sham	3.12 ± 0.12	35.71 ± 3.10	1.50 ± 0.50
OA	4.03 ± 0.35 ^a^	64.30 ± 12.49 ^a^	3.88 ± 0.18 ^a^
Diclofenac	3.40 ± 0.11 ^ac^	47.36 ± 7.24 ^ac^	2.74 ± 0.51 ^ac^
Mixed formula—PCP:EC:AR			
200 mg/kg	3.24 ± 0.08 ^bc^	41.73 ± 4.17 ^ac^	2.36 ± 0.39 ^ac^
100 mg/kg	3.42 ± 0.07 ^ac^	47.31 ± 7.99 ^ad^	2.80 ± 0.51 ^ac^
50 mg/kg	3.66 ± 0.16 ^ac^	50.63 ± 3.79 ^ac^	3.40 ± 0.30 ^ad^

Values are expressed mean ± SD of eight rabbits, μg/L. OA = Osteoarthritis; AR = Aqueous extracts of Achyranthis Radix; EC = Aqueous extracts of Eucommiae Cortex; PCP = Dried Pomegranate Juice Concentrated Powder; COMP = Cartilage oligomeric matrix protein; CTX-II = Collagen type II C-telopeptide; MMP = Matrix metalloproteinase. ^a^ *p* < 0.01 and ^b^ *p* < 0.05 as compared with sham control; ^c^ *p* < 0.01 and ^d^ *p* < 0.05 as compared with OA control.

**Table 4:** Synovial COMP, CTX-II, MMP-3, IL-1β, and TNF-α content in sham-operated or OA rabbits.

Items Groups	Synovial Contents (μg/L)				
	COMP	CTX-II	MMP-3	IL-1β	TNF-α
Controls					
Sham	5.24 ± 0.47	123.56 ± 22.90	4.71 ± 0.54	13.39 ± 2.95	7.37 ± 1.95
OA	37.20 ± 5.85 ^a^	325.50 ± 50.20 ^a^	39.94 ± 2.44 ^a^	88.77 ± 14.63 ^a^	75.85 ± 8.93 ^a^
Diclofenac	24.94 ± 3.61 ^ab^	206.73 ± 51.26 ^ab^	26.07 ± 4.34 ^ab^	58.00 ± 10.89 ^ab^	45.49 ± 11.00 ^ab^
Mixed formula—PCP:EC:AR					
200 mg/kg	17.65 ± 4.37 ^ab^	171.19 ± 42.48 ^b^	22.25 ± 5.05 ^ab^	37.52 ± 11.91 ^ab^	30.04 ± 10.42 ^ab^
100 mg/kg	25.56 ± 4.15 ^ab^	216.05 ± 23.29 ^ab^	26.96 ± 4.46 ^ab^	56.49 ± 10.86 ^ab^	44.04 ± 13.89 ^ab^
50 mg/kg	30.93 ± 2.95 ^ab^	245.86 ± 25.75 ^ab^	31.85 ± 5.89 ^ab^	65.98 ± 10.93 ^ab^	55.71 ± 13.52 ^ab^

Values are expressed mean ± SD of eight rabbits, μg/L. OA = Osteoarthritis; AR = Aqueous extracts of Achyranthis Radix; EC = Aqueous extracts of Eucommiae Cortex; PCP = Dried Pomegranate Juice Concentrated Powder; COMP = Cartilage oligomeric matrix protein; CTX-II = Collagen type II C-telopeptide; MMP = Matrix metalloproteinase; IL = Interleukin; TNF = Tumor necrosis factor. ^a^ *p* < 0.01 as compared with sham control; ^b^ *p* < 0.01 as compared with OA control.

**Table 5:** Femur and tibia articular cartilage Mankin scores in sham-operated or OA rabbits.

Groups Items	Control		Diclofenac	Mixed Formula—PCP:EC:AR		
	Sham	OA		200 mg/kg	100 mg/kg	50 mg/kg
Femur						
Surface	0.50 ± 0.53	3.00 ± 0.00 ^a^	1.75 ± 0.46 ^ac^	0.75 ± 0.46 ^c^	1.88 ± 0.64 ^ac^	2.25 ± 0.46 ^ad^
Cellularity	0.25 ± 0.46	2.63 ± 0.52 ^a^	1.63 ± 0.52 ^ac^	0.63 ± 0.52 ^c^	1.75 ± 0.46 ^ac^	1.88 ± 0.83 ^ad^
Clone	0.00 ± 0.00	2.75 ± 0.46 ^a^	1.13 ± 0.83 ^bc^	0.63 ± 0.52 ^bc^	1.50 ± 0.76 ^ac^	1.88 ± 0.83 ^ad^
Stain intensity	0.38 ± 0.52	2.00 ± 0.76 ^a^	1.50 ± 0.52 ^a^	1.00 ± 0.76 ^c^	1.63 ± 0.52 ^a^	2.13 ± 0.64 ^a^
Total *	1.13 ± 0.83	10.83 ± 1.30 ^a^	6.00 ± 1.07 ^ac^	3.00 ± 1.31 ^ac^	6.75 ± 1.75 ^ac^	8.13 ± 1.55 ^ac^
Tibia						
Surface	0.38 ± 0.52	2.50 ± 0.53 ^a^	1.00 ± 0.76 ^c^	0.88 ± 0.83 ^c^	1.50 ± 0.53 ^ac^	2.25 ± 0.46 ^a^
Cellularity	0.50 ± 0.53	2.50 ± 0.53 ^a^	1.50 ± 0.53 ^bd^	0.63 ± 0.52 ^c^	1.13 ± 0.35 ^c^	1.63 ± 0.52 ^ad^
Clone	0.00 ± 0.00	2.13 ± 0.83 ^a^	1.13 ± 0.83 ^bd^	0.50 ± 0.53 ^c^	1.00 ± 0.00 ^ad^	1.38 ± 0.92 ^a^
Stain intensity	0.38 ± 0.52	2.13 ± 0.64 ^a^	1.00 ± 0.76 ^bc^	0.75 ± 0.46 ^c^	1.13 ± 0.35 ^bc^	1.75 ± 0.71 ^a^
Total *	1.25 ± 1.28	9.25 ± 1.04 ^a^	4.63 ± 1.60 ^ac^	2.75 ± 1.28 ^bc^	4.75 ± 0.89 ^ac^	7.00 ± 1.41 ^ac^

Values are expressed mean ± SD of eight rabbits, score (* Max of totalized scores = 12). OA = Osteoarthritis; AR = Aqueous extracts of Achyranthis Radix; EC = Aqueous extracts of Eucommiae Cortex; PCP = Dried Pomegranate Juice Concentrated Powder. ^a^ *p* < 0.01 and ^b^ *p* < 0.05 as compared with sham control; ^c^ *p* < 0.01 and ^d^ *p* < 0.05 as compared with OA control.

**Table 6:** Femur and tibia articular cartilage general histomorphometrical analysis in sham-operated or OA rabbits.

Groups Items	Control		Diclofenac	Mixed Formula—PCP:EC:AR		
	Sham	OA		200 mg/kg	100 mg/kg	50 mg/kg
Femur articular cartilage						
Thickness ^1^	1372.82 ± 175.61	531.29 ± 108.43 ^a^	892.33 ± 144.88 ^ab^	1025.15 ± 188.58 ^ab^	858.59 ± 104.91 ^ab^	738.97 ± 90.30 ^ab^
Chondrocytes ^2^	494.88 ± 69.50	95.63 ± 23.49 ^a^	204.63 ± 38.14 ^ab^	296.00 ± 27.56 ^ab^	192.88 ± 32.95 ^ab^	136.63 ± 23.38 ^ab^
Subchondral IL-1β ^2^	23.38 ± 5.53	361.25 ± 70.56 ^a^	88.88 ± 25.91 ^ab^	65.88 ± 19.25 ^ab^	113.13 ± 18.59 ^ab^	214.00 ± 47.99 ^ab^
Subchondral TNF-α ^2^	17.25 ± 4.23	429.00 ± 100.94 ^a^	143.63 ± 43.55 ^ab^	76.50 ± 17.36 ^ab^	143.13 ± 19.86 ^ab^	275.38 ± 71.06 ^ab^
Tibia articular cartilage						
Thickness ^1^	1896.66 ± 176.58	577.70 ± 108.46 ^a^	866.53 ± 157.81 ^ab^	1105.09 ± 182.50 ^ab^	845.26 ± 170.82 ^ab^	728.59 ± 64.47 ^ac^
Chondrocytes ^2^	328.75 ± 61.26	71.50 ± 14.63 ^a^	136.38 ± 29.69 ^ab^	218.88 ± 37.52 ^ab^	126.63 ± 37.75 ^ab^	100.63 ± 18.19 ^ab^
Subchondral IL-1β ^2^	24.38 ± 10.93	270.88 ± 25.39 ^a^	110.63 ± 32.63 ^ab^	102.75 ± 21.99 ^ab^	127.25 ± 23.09 ^ab^	215.13 ± 35.06 ^ab^
Subchondral TNF-α ^2^	38.50 ± 12.80	309.00 ± 44.82 ^a^	110.13 ± 26.94 ^ab^	81.00 ± 11.76 ^ab^	134.88 ± 26.44 ^ab^	219.88 ± 49.99 ^ab^

Values are expressed mean ± SD of eight rabbits. ^1^ μm; ^2^ cells/mm^2^. OA = Osteoarthritis; AR = Aqueous extracts of Achyranthis Radix; EC = Aqueous extracts of Eucommiae Cortex; PCP = Dried Pomegranate Juice Concentrated Powder; AC = Articular cartilage; IL = Interleukin; TNF = Tumor necrosis factor. ^a^ *p* < 0.01 as compared with sham control; ^b^ *p* < 0.01 and ^c^ *p* < 0.05 as compared with OA control.

## Supplementary Materials

The following are available online at https://www.mdpi.com/2072-6643/12/3/852/s1, Figure S1: Representative X-ray images of the knee joints, taken from sham-operated or OA rabbits.
