# Supplementary material for: Anti-Osteoarthritic Effects of a Mixture of Dried Pomegranate Concentrate Powder, Eucommiae Cortex, and Achyranthis Radix 5:4:1 (g/g) in a Surgically Induced Osteoarthritic Rabbit Model
Source: Nutrients. 2020 Mar 22;12(3):852. doi: 10.3390/nu12030852 (PMC7146119; doi:10.3390/nu12030852)
Supplement: Supplementary file 1 [file nutrients-12-00852-s001.pdf]

Supplementary Materials:

1. Materials and Methods

1. Roentgenographic examination

Fifty-six days after the initial treatment administration, all rabbits underwent roentgenographic examination to assess chronic morphological changes in the knee bones, such as narrowing, loss of the joint region, cartilage erosion, or osteophyte formation (in accordance with previous studies) [1,2] using an automated digital roentgenographic system (Carestream DRX-1 system; Rochester, NY, USA) with X-ray equipment (CS-60; Dong Kang Medical systems, Pyeongtaek, Korea).

2. Result

1. Effects of treatment seen on roentgenographic images

OA operated rabbits showed narrowing of the articular space, loss of the knee joint region, femoral and tibial AC erosion, and osteophyte formation. However, dose-dependent decreases in OA-like X-ray signs were s noted in all PCP:EC:AR 5:4:1 (g/g) (200, 100, and 50 mg/kg)-treated rabbits compared with the OA control rabbits (Fig S1).

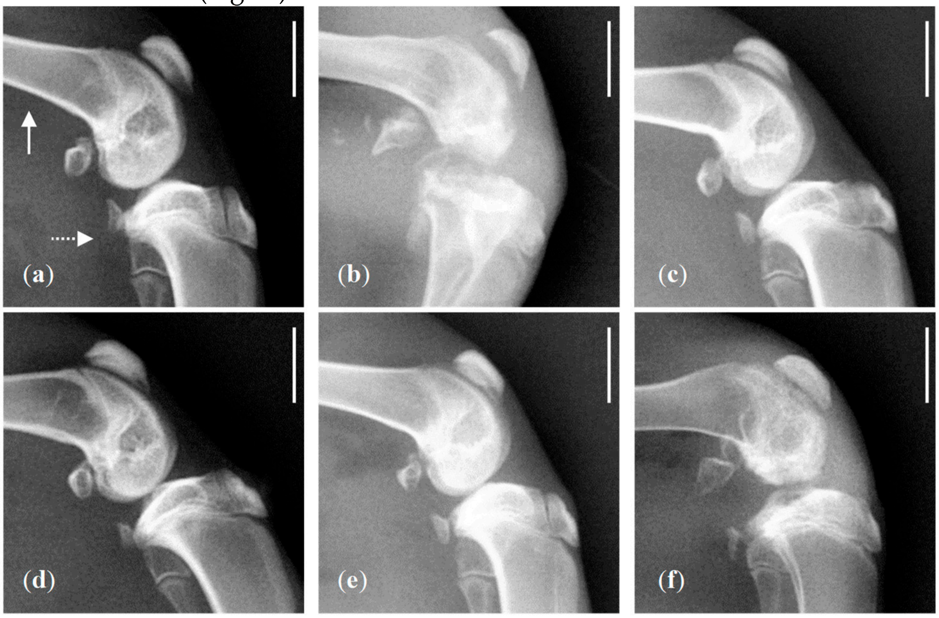

Figure S1. Representative X-ray images of the knee joints, taken from sham-operated or OA rabbits. (**a**) Sham vehicle control (sham-operated and distilled water orally administered rabbits); (**b**) OA control (OA-surgery and distilled water orally administered rabbits); (**c**) Diclofenac (OA-surgery and diclofenac sodium 2 mg/kg subcutaneously treated rabbits); (**d**) PCP:EC:AR 5:4:1 (*g/g*) mixed formula 200 mg/kg orally administrated OA rabbits; (**e**) PCP:EC:AR 5:4:1 (*g/g*) mixed formula 100 mg/kg orally administrated OA rabbits; (**f**) PCP:EC:AR 5:4:1 (*g/g*) mixed formula 50 mg/kg orally administrated OA rabbits. OA = Osteoarthritis; AR = Aqueous extracts of Achyranthis Radix; EC = Aqueous extracts of Eucommiae Cortex; PCP = Dried Pomegranate Juice Concentrated Powder. Arrows = Femur; Dot arrows = Tibia. Scale bars = 1 cm.

1. Sakano, Y.; Terada, N.; Ueda, H.; Fujii, Y.; Hamada, Y.; Akamatsu, N.; Ohno, S. Histological study of articular cartilage in experimental rat knee arthritis induced by intracapsular injection of cationic polyethyleneimine. Med Electron Microsc 2000, 33, 246-257, doi:10.1007/s007950000025.
2. Kim, J.K.; Park, S.W.; Kang, J.W.; Kim, Y.J.; Lee, S.Y.; Shin, J.; Lee, S.; Lee, S.M. Effect of GCSB-5, a Herbal Formulation, on Monosodium Iodoacetate-Induced Osteoarthritis in Rats. Evid Based Complement Alternat Med 2012, 2012, 730907, doi:10.1155/2012/730907.
